# The effects of exercise training versus intensive insulin treatment on skeletal muscle fibre content in type 1 diabetes mellitus rodents

**DOI:** 10.1186/s12944-021-01494-w

**Published:** 2021-07-06

**Authors:** David P. McBey, Michelle Dotzert, C. W. J. Melling

**Affiliations:** 1 School of Kinesiology, Western University, Medical Sciences Building 227, London, ON N6A 3K7 Canada; 2 Department of Physiology and Pharmacology, Schulich School of Medicine, Western University, London, ON Canada

**Keywords:** Type 1 diabetes mellitus, Exercise, Skeletal muscle fibre, Intramyocellular lipids, Muscle glycogen, Insulin treatment

## Abstract

**Background:**

Intensive-insulin treatment (IIT) strategy for patients with type 1 diabetes mellitus (T1DM) has been associated with sedentary behaviour and the development of insulin resistance. Exercising patients with T1DM often utilize a conventional insulin treatment (CIT) strategy leading to increased insulin sensitivity through improved intramyocellular lipid (IMCL) content. It is unclear how these exercise-related metabolic adaptations in response to exercise training relate to individual fibre-type transitions, and whether these alterations are evident between different insulin strategies (CIT vs. IIT). Purpose: This study examined glycogen and fat content in skeletal muscle fibres of diabetic rats following exercise-training.

**Methods:**

Male Sprague-Dawley rats were divided into four groups: Control-Sedentary, CIT- and IIT-treated diabetic sedentary, and CIT-exercised trained (aerobic/resistance; DARE). After 12 weeks, muscle-fibre lipids and glycogen were compared through immunohistochemical analysis.

**Results:**

The primary findings were that both IIT and DARE led to significant increases in type I fibres when compared to CIT, while DARE led to significantly increased lipid content in type I fibres compared to IIT.

**Conclusions:**

These findings indicate that alterations in lipid content with insulin treatment and DARE are primarily evident in type I fibres, suggesting that muscle lipotoxicity in type 1 diabetes is muscle fibre-type dependant.

**Supplementary Information:**

The online version contains supplementary material available at 10.1186/s12944-021-01494-w.

## Background

Type I diabetes mellitus (T1DM) is an autoimmune-related disorder characterized by the destruction of insulin-producing beta cells in the pancreatic islets of Langerhans. This results in chronically low levels of circulating insulin and the subsequent loss of glycemic control. The inability to maintain blood glycemic equilibrium can result in elevated blood glucose (BG) levels, cumulating in a condition known as hyperglycemia. Chronic hyperglycemia can result in the development of cardiovascular disease (CVD), retinopathy, neuropathy, nephropathy, and myopathy, which represent the greatest contributors to morbidity and mortality for patients with T1DM [1–3]. Despite the many advances made in T1DM research, global prevalence of the disease has continued to increase particularly in youth populations [2, 4]. By 2018, T1DM was one of the most frequently diagnosed chronic diseases in children and youth, and global prevalence is expected to continue to rise [5].

Traditionally, T1DM was managed using conventional insulin therapy (CIT). This strategy consists of a bolus of insulin taken once or twice daily, coupled with daily urine or BG testing. The modern treatment strategy, known as intensive insulin therapy (IIT), requires BG monitoring throughout the day, adjusting diet and insulin dosage with the goal of maintaining BG as close to the normal range as possible. With the publication of the Diabetes Control and Complications Trial in 1993, the evidence supports a significant long-term benefit of IIT over CIT [3]. Specifically, IIT leads to significant reductions in the long-term development of CVD (42%), retinopathy (76%), neuropathy (69%), and nephropathy (34%), in T1DM cases [6–12]. However, in approximately 20% of these cases, long-term reliance on IIT has been associated with the development of insulin resistance (IR), a phenomenon often associated with type 2 diabetes mellitus (T2DM) [13]. Termed “double diabetes”, these patients are at an increased risk for CVD and other associated morbidities when compared to patients with either T1DM or T2DM alone [14, 15]. As muscle tissue is the final destination for approximately 80–90% of circulating BG in healthy and T2DM patients, muscle IR can be highly disruptive of glucose homeostasis [16–19]. While it has been suggested that the development of IR in these populations has been due to impaired intramuscular glycogen synthesis [17], it has recently been recognized that the presence of IR in T1DM is pathologically different from other conditions like T2DM, though the IR pathogenesis in this already high-risk population remains unknown [15, 20].

One promising theory underlying the development of “double diabetes” in T1DM is the muscle-lipotoxicity theory of IR [7, 13, 21, 22]. This theory posits that the improper storage of intramyocellular lipid (IMCL) metabolites in skeletal muscle leads to disruption of the insulin receptor signalling pathway. The result of this impaired insulin response is the consequent development of chronic hyperglycemia [7]. It is believed that the onset of double diabetes begins with inadequate levels of circulating insulin, which results in an increase in muscle lipid flux that the mitochondria are unable to metabolize. This “metabolic overload” ultimately results in the conversion of free fatty acids into diacylglycerol (DAG) or metabolization into ceramides [23]. The resulting accumulation of these IMCLs initiates the development of IR, as both DAG and ceramides have been shown to interfere with the insulin signalling pathway [24–26] and have been positively correlated with IR severity [13].

Most research regarding muscular adaptations to T1DM have included markers of metabolic function gathered from whole skeletal muscle. While these papers demonstrate that T1DM leads to significant decreases in muscle oxidative capacity [4, 27–30], only a few studies have examined differential changes in metabolic function between each distinct muscle fibre type [31]. In T1DM skeletal muscle, type I slow-twitch oxidative fibres exhibit significantly reduced citrate synthase activity, a marker for oxidative phosphorylation which is often associated with changes in mitochondrial morphology [31–33]. Type IIa fast-twitch oxidative fibres display a combination of reduced enzymatic activity for both oxidative and glycolytic processes in T1DM muscle [31, 32, 34]. Type IIb fast-twitch glycolytic muscle fibres express reduced enzymatic activity primarily for markers of glycolytic flux, including hexokinase, phosphofructokinase-1, and phosphatase [31]. Since type IIb fibres specifically rely primarily on glycolytic rather than oxidative metabolism, the T1DM-related inhibition of oxidative capacity has a reduced effect on type IIb fibres. However, the induction of T1DM does lead to a preferential reduction in type IIb fibre cross-sectional area, resulting in reduced overall skeletal muscle mass [27, 35, 36]. Other indicators of glycolytic activity such as glycerol-3-phosphate dehydrogenase have previously been shown to increase across all fibre types in patients with T1DM, suggesting that despite differences in individual fibres, whole-muscle adaptations may trend away from oxidative and towards increased glycolytic flux in T1DM muscle [33].

The majority of studies examining exercise training in T1DM have focused primarily on improving indices of glycemic control, and it has been discovered that a combination of resistance and aerobic exercise provides the greatest metabolic benefits while reducing the incidence of post-exercise hypoglycemia, which is a primary concern when exercising with T1DM [10, 37–44]. There is also evidence that the training adaptations from resistance and aerobic exercise may complement each other, leading to improved metabolic markers when compared to resistance or aerobic exercise alone [22, 37, 38, 45]. Citrate synthase activity and muscle IMCL content, for example, were shown to increase in a combined exercise-trained T1DM muscle when compared to aerobic exercise alone, suggesting that combined training may also improve the muscle’s ability to effectively store and oxidize IMCL [22]. Moreover, these improvements are evident in patients with T1DM who altered their insulin regimen and intentionally elevated BG to levels that represent the more traditional CIT strategy in order to mitigate hypoglycemia onset [10, 37, 39, 46–49].

Research is needed to investigate the specific transitional and metabolic changes to muscle fibre profile resulting from combined resistance and aerobic exercise (with CIT) in T1DM skeletal muscle. Studies are needed to explore exercise-related changes in skeletal muscle morphology in comparison to IIT, the modern standard of care for patients with T1DM. The purpose of this study was to examine the effects of a combined exercise training regimen (with CIT) versus IIT on the skeletal muscle fibre profile of T1DM rodents. Specifically, this study investigated whether combined exercise led to a transition towards more oxidative type I fibres, concomitant with increased IMCL content and stored muscle glycogen content. Firstly, it was hypothesized that a regimen of IIT would cause a shift in muscle fibre type towards a higher percentage of type IIb fibres with high IMCL stores. Secondly, it was hypothesized that a combined aerobic and resistance exercise training regimen (with CIT) would oppose this change, leading to a reduction in IMCL stores in type IIb fibres, and a shifting of fibre type towards an oxidative type I and IIa fibre profile.

## Methods

### Ethics approval

The University Council of Animal Care of Western University (London, Ontario, Canada) approved the protocols in this study by in accordance with the standards of the Canadian Council on Animal Care.

### Animals

In advance of the study, a power calculation to determine group size returned an appropriately powered sample size of *n* = 5 animals for each of four groups (based on a power of 80% and an alpha value of 0.05). Therefore, twenty male Sprague-Dawley rats were acquired from Charles River Laboratories (St. Constant, Quebec, Canada) at the age of 8 weeks. The rats were caged in pairs and held on a consistent 12-h dark/light cycle, with room temperature held at 20 ± 1 °C and relative humidity at 50% for the duration of the study. All rats were given access to standard rat chow and water ad libitum throughout the study.

### Experimental groups

Each rat was randomly assigned into 1 of 4 treatment groups: control sedentary (CS; *n* = 4), diabetic sedentary treated with CIT (DCT; *n* = 4), diabetic sedentary treated with IIT (DIT; *n* = 5), and diabetic with combined exercise training, treated with CIT (DARE; *n* = 4).

While each of the groups began the study with a similar number of animals (*n* = 5), three of the four groups had one animal each that experienced negative reactions to the anaesthetics used during the surgical procedure to alter insulin dosage (subcutaneous insulin pellets were implanted and adjusted throughout the study to maintain blood glucose levels in the desired ranges). Consequently, these three animals were removed from the study and sacrificed.

### Experimental procedures

#### T1DM induction and insulin pellet implantation

Following an acclimatization period of 5 days, the rats in the three diabetes groups were given an intraperitoneal low dose injection of 20 mg/kg streptozotocin (STZ; Sigma-Aldrich Canada Co., Oakville, ON, Canada) every day for 5 days, in order to induce T1DM. All STZ injections were given within 5 min of preparation, dissolved in a 0.1 M citrate buffer (pH 4.5). T1DM was confirmed using non-fasted BG measurements of ^3^18 mmol/L on two consecutive days. Thereafter, once T1DM had been confirmed, abdominal subcutaneous insulin pellets were surgically implanted into the three diabetic groups of rats receiving insulin therapy (1 pellet; 2 U insulin/day; Linplant, Linshin). The insulin pellet therapy was altered during the course of the experiment through the addition or removal of insulin pellet amounts, so that BG ranges reflected the tightly monitored and moderately hyperglycemic conditions that are more representative of the general human population living with T1DM: DCT rats maintained a BG range of 9–15 mmol/L [3, 50], DIT at 7–9 mmol/L [3, 47, 50], and DARE at 9–15 mmol/L [39].

#### Exercise training protocols

Rats in the DARE experimental group became familiarized with the exercise equipment over the course of a week, then underwent a combined regimen of aerobic and resistance exercise training throughout the next 12 weeks. Combined resistance and aerobic exercise training involved alternating 5 days per week for 12 weeks, where the first week would have resistance (R) training performed 3 days of the week, alternating with 2 days of high-intensity aerobic (A) exercise (R-A-R-A-R), followed by a week of the opposite (with 3 days of aerobic exercise alternating with 2 days of resistance exercise; A-R-A-R-A).

Familiarization with the aerobic exercise protocol involved running for 15 min at progressively higher speeds on a motorized treadmill (up to 30 m/min at a 0° incline) at five and 3 days before the start of the training program. The high intensity aerobic training program involved DARE rats running for 1 h at 27 m/min on a 6% incline gradient, eliciting an exercise intensity of between 70 and 80% of their maximum rate of oxygen consumption (VO_2_max) [51]. The rats were encouraged to maintain pace by short bursts of compressed air triggered when they slowed and broke a photoelectric beam at the back of the treadmill.

Resistance exercise training incorporated the use of a weighted ladder climb on alternating days with the aerobic training. At the top of the ladder was an open, dark box where the rats could shelter. Weights were placed into fabric bags attached to the base of their tails and the rats were then allowed to climb up the ladder. This process was repeated with increasing weights as the rats adapted to each weight load, in order to maintain appropriate stimulus to the muscle so as to reproduce a resistance training protocol. Familiarization involved 10 ladder climbs with progressive weight increases up to 35% of body mass at 5 and 3 days before the onset of training. Following each climb, the rats were permitted a 2-min rest in the box. ﻿Pre-training maximal carrying capacity was determined by initial loading of 75% body mass, with each subsequent climb adding 30 g of weight until animals were unable to successfully complete the climb to the preferred dark box destination at the top of the ladder. During training, the DARE rats were loaded with 50, 75, 90, and 100% of their predetermined maximal carrying capacity for single climbs, and then repeated 100% load climbs until exhaustion or unwillingness to climb despite encouragement via pressurized air bursts. Maximal carrying capacity was re-evaluated every four exercise sessions, ensuring the rats were undergoing a progressive resistance exercise regime.

### Experimental measures

#### Body weights, blood glucose

Body weights were measured and recorded weekly throughout the course of the study. The implanted insulin pellets issued ~ 2 doses per day at a constant release rate, and non-fasted BG readings were taken twice weekly at 9 am by collecting a small droplet of blood (~ 50 μL) from the saphenous vein throughout the duration of the study. BG values were analyzed via a Freestyle Lite Blood Glucose Monitoring System (Abbott Diabetes Care, INC. Conyers, GA, USA) and reported in millimoles per litre (mmol/L).

#### Tissue collection

Rats were sacrificed at 10:00 am, 72 h following their last training session, via anaesthetization with isoflurane, followed by cardiac exsanguination. Lower limbs were dissected and the plantaris muscles were removed, mounted in ﻿Cryomatrix™ embedding medium (Lot No. 225229, Thermo Fisher Scientific, Waltham, MA, USA) and rapidly frozen in isopentane cooled to − 70 °C by liquid nitrogen. Plantaris was chosen due to its 50:50 mix of red and white muscle tissue, with the intention of highlighting any changes to both oxidative and glycolytic fibre types. Serial cryosections were cut in varying thicknesses (12 μm:gPAS, 12 μm:OilRedO, 10 μm:mATPase) at − 20 °C with a Leica CM350 Cryostat (Leica Biosystems, Concord, ON, Canada) and adhered to VWR Superfrost® Plus Microslides (Cat. No. 48311–703, VWR International, Mississauga, ON, Canada). All sections were stored at − 30 °C until analysis.

### Histochemical analysis

#### Metachromatic myosin ATPase for muscle fibre type analysis

**Fig. 1:**
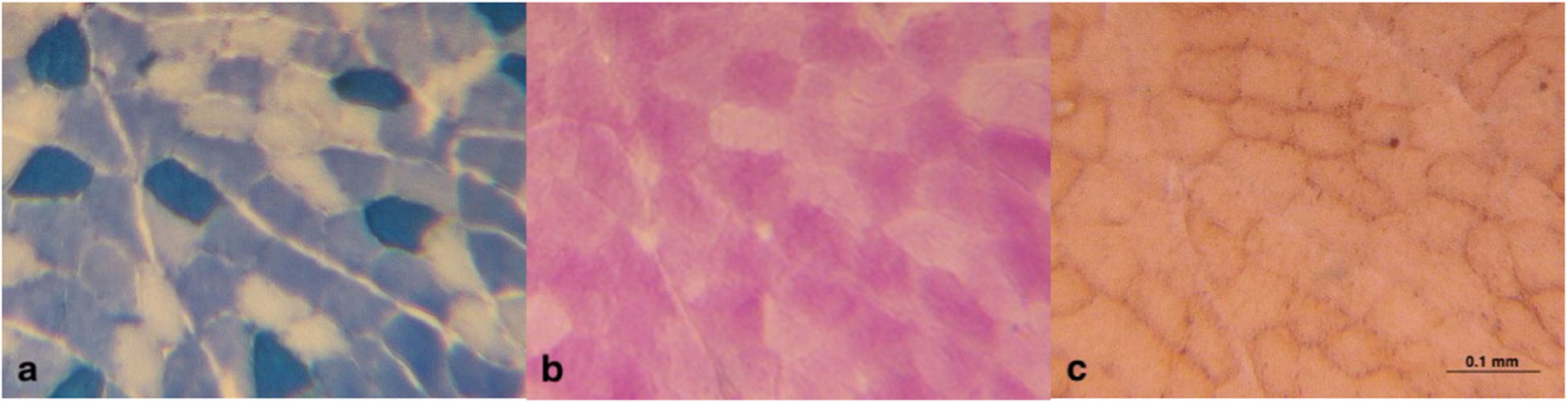
Examples of serial sections with three histochemical stains used in fibre quantification, from left to right: **a** Metachromatic myosin ATPase (stain to identify muscle fibre type), **b** Glycogen Periodic Acid Schiff (stain to identify high/low glycogen content), and **c** Oil Red O (stain for high/low IMCL content). See Additional file 1 for quantification example. Note that Oil Red O-stained neutral lipids appear to primarily exist around the internal periphery of the cell membrane, suggesting a possible role for subcellular localization of lipid droplets in the utility of IMCL

Slides were treated using the metachromatic dye-based myosin ATPase stain protocol initially developed by Ogilvie et al., adapted for use in rat muscle tissue by Walsh et al. [52, 53]. Slides were incubated in an acidic pre-incubation solution (pH 4.38) for 7 min, followed by 3 × 2 min washes in Tris buffer (pH 7.8). Slides were then incubated for 30 min at room temperature in an adenosine triphosphate (ATP) incubation solution (pH 9.4). After incubation, slides were dipped 4x in 3 changes of a CaCl_2_-2H_2_O solution, then incubated for 90 s in a 0.1% toluidine blue solution before being rinsed in running ddH_2_O to clear excess stain. Finally, slides were dehydrated in ascending alcohols, cleared with xylenes, and mounted with a toluene-based mounting medium. Type I muscle fibres stained dark blue, type IIa fibres stained white, and type IIb fibres stained light blue (Fig. 1a).

#### Glycogen periodic acid Schiff for glycogen staining

Slides were placed directly in Carnoy’s fixative (ethanol, chloroform + glacial acetic acid) at − 30 °C for 5–10 min, then continued incubation while returning to room temperature. Carnoy’s fixative was discarded and slides were rinsed in 3 changes of ddH_2_O, before being placed in 0.5% periodic acid for 5 min. Slides were left to incubate in 37.5 °C Schiff’s reagent for 30 min, then cleared with running 37 °C tap water for 10 min. Slides were dehydrated in ascending alcohols, cleared with xylenes, and mounted with toluene-based mounting media. Fibres containing high glycogen stores stained a bright pink/purple, while those with low glycogen stores stained white (Fig. 1b).

#### Oil red O for quantification of neutral lipids

Muscle sections stained following the protocol described by Dotzert et al. [13]. Fibres with high IMCL stores stained dark reddish/brown, while those with low IMCL stores were a light-pink-ish/yellow colour in comparison (Fig. 1c).

### Microscopy image collection and muscle fibre analysis

All microscopic imaging was performed on a Zeiss AxioVert S100 microscope equipped with a Canon EOS Rebel T2i camera adapted for use with an NDPL-1(2x) attachment. Muscle sections were captured at 10x magnification in a grid pattern so that each region of each slide was photographed, allowing the same region to be compared between each of the histochemical slides.

The grid photographs were quantified, ensuring the same fibres were examined across each stain. Fibre numbers were tallied so that a minimum of 200 fibres were included per stain per animal to ensure samples would provide accurate representation of the muscle fibre profile as a whole, with each of the fibres containing data from four different stains (Hematoxylin & Eosin, fibre type, fat content, glycogen content). High/low IMCL/glycogen storage content and muscle fibre identity were evaluated based on staining colour specific to each procedure. Muscle fibres were only included if they were clearly defined across all stains, with a clear distinction between those fibres containing high IMCL/glycogen stores versus those with low stores, and clear distinction between fibre type, so as to avoid incomplete or imprecise analysis (see Additional file 1 for example). An independent analysis was performed by a blinded third-party analyst, and similar results were obtained (average 2.5% difference across quantification categories, 221 fibres).

### Data analysis

Muscle fibre type, fibre glycogen content, and fibre IMCL content were compared within and between groups using a one-way analysis of variance (ANOVA) with GraphPad Prism 6 (GraphPad Software, Inc., San Diego, CA, USA). When significant differences were observed, pairwise post-hoc analysis was performed using the Tukey test. For results demonstrating a significant Brown-Forsythe test for equality of group variances, post-hoc analysis was performed using the Welch’s ANOVA for unequal variances. Significance was determined with a confidence interval of 95% for an alpha value of 0.05.

## Results

### Animal characteristics

**Fig. 2:**
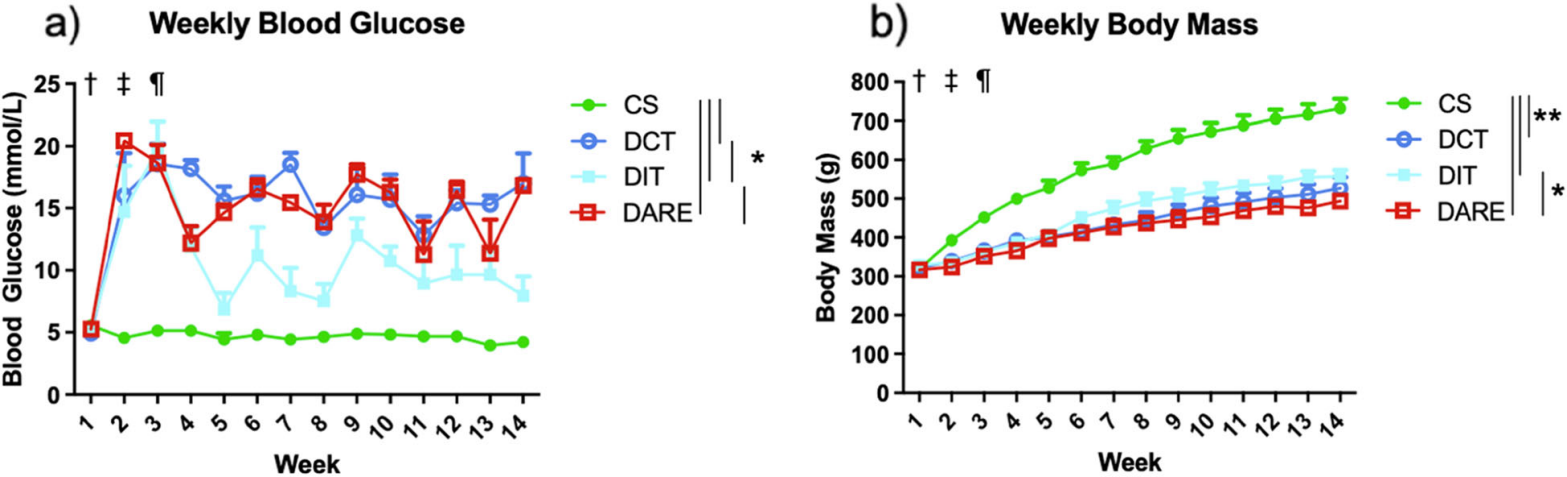
Complete blood glucose and body weight data are presented in (**a**) and (**b**), respectively. These data were collected for 17 male rats across four groups throughout the duration of the study**.** † denotes the start of streptozotocin injections to induce type 1 diabetes; ‡ denotes the start of insulin treatment; and ¶ denotes the start of exercise regimen. **a** Mean non-fasted weekly BG measures (mmol/L). All data presented as mean ± SEM. * denotes *P* ≤ 0.0001. Insulin was administered through subcutaneous implants, and pellet dosage was adjusted throughout the study to maintain desired blood glucose ranges **b** Mean weekly body mass measures (g). All data presented as mean ± SEM. * denotes *P* = 0.0059, ** denotes *P* < 0.0001. The four groups compared were Control Sedentary (CS); diabetic with conventional insulin therapy (DCT); diabetic with intensive insulin therapy (DIT); and diabetic with combined exercise training and conventional insulin therapy (DARE). Note that the body mass of all three diabetic groups was significantly lower than the mass of the CS group (*P* < 0.0001)

Animal physical characteristics included weekly blood glucose BG (mmol/L) and weekly body mass (g) measures. Complete body weight and BG data were collected for 17 animals (CS *n* = 4; DCT *n* = 4; DIT *n* = 5; DARE *n* = 4). These results are presented in Fig. 2a and b*,* respectively.

Weekly glucose measures were significantly different between all groups (*P* ≤ 0.0001) except for DCT vs. DARE, for which no significant difference was found (*P* > 0.05; Fig. 2a*)*.

Weekly body mass analysis identified a significant difference between the DIT and DARE groups (*P* < 0.01), although no other significant differences between the diabetic groups (*P* > 0.05; DCT, DIT, and DARE) were identified. The body mass for all three diabetic groups was significantly lower than the mass of the CS animals (*P* < 0.0001) (Fig. 2b*)*.

### Type I fibres: IMCL stores, muscle glycogen stores, and total muscle percentage

**Fig. 3:**
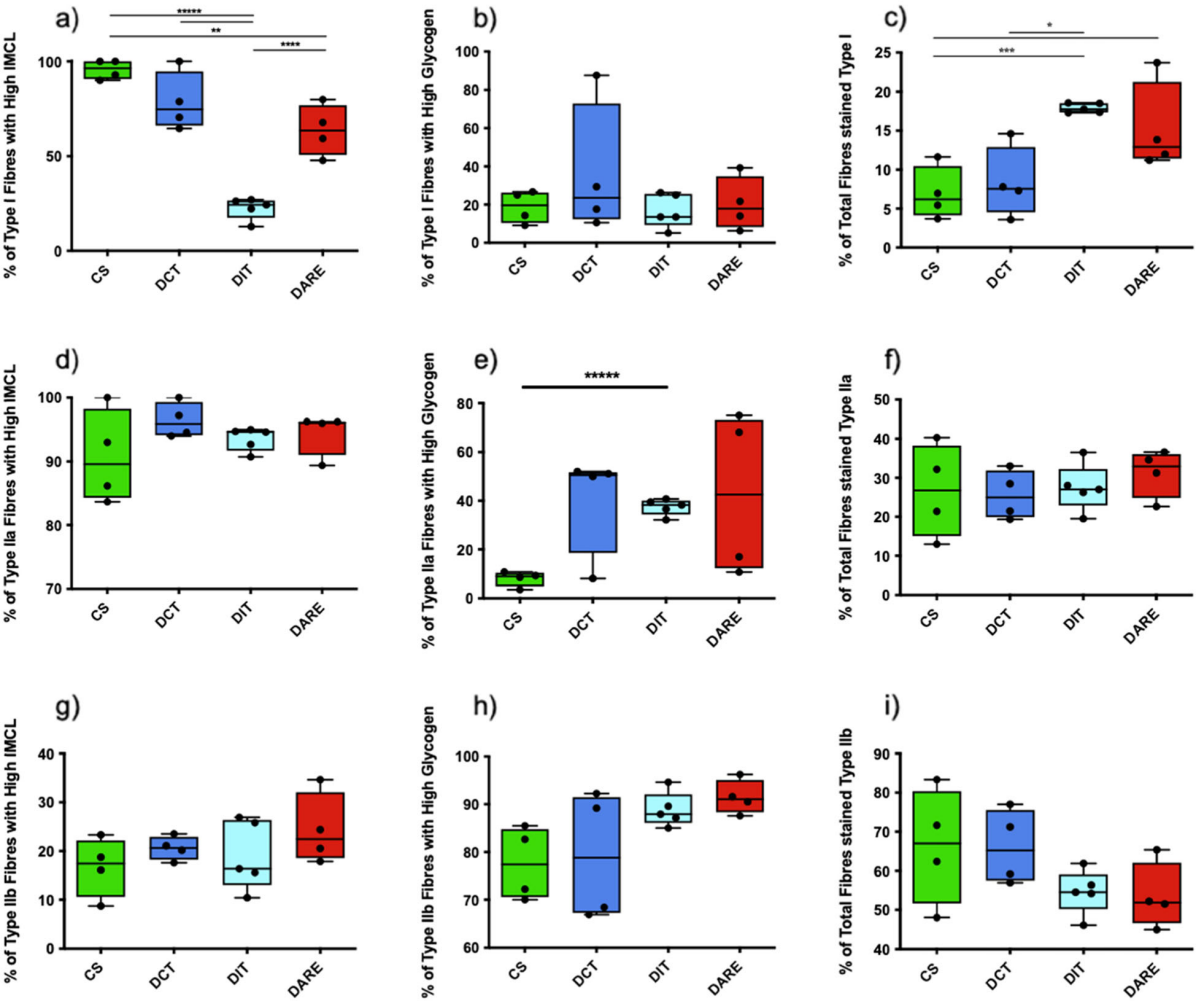
The fibre-type specific metabolic profiles of 3630 muscle fibres from 17 male rats across four groups. The top, middle, and bottom rows present data for type I (**a-c**), type IIa (**d-f**), and type IIb myofibres (**g-h**) respectively. The columns from left to right present data on the percentage of high neutral-IMCL containing fibres (as assessed via Oil Red O staining) (**a,d,g**), the percentage of high-glycogen containing fibres (as assessed via glycogen periodic acid Schiff staining) (**b,e,h**), and finally the overall percentage of each row’s muscle fibre as a percentage of the total fibres quantified (fibre type assessed via metachromatic myosin ATPase staining) (**c,f,i**). The four groups are Control Sedentary (CS); diabetic with conventional insulin therapy (DCT); diabetic with intensive insulin therapy (DIT); and diabetic with combined exercise training and conventional insulin therapy (DARE). All data are expressed as mean ± SEM. * denotes 0.05 > *P* > 0.005; ** denotes *P* = 0.0047; *** denotes *P* = 0.0017; **** denotes *P* = 0.0003; ***** denotes *P* < 0.0001. Note that all changes in IMCL storage occurred within the type I oxidative fibres, and that there are a greater percentage of these type I myofibres in the DIT and DARE groups when compared to control and DCT

Rats in the DIT group had a significantly lower percentage of high-IMCL type I fibres than animals in the CS (*P* < 0.0001), DCT (*P* < 0.0001) and DARE groups (*P* < 0.001) (Fig. 3a*)*. There was a significantly smaller percentage of high-IMCL type I fibres in the DARE group compared to CS as well (*P* < 0.005; Fig. 3a*).*

No significant differences were observed between all groups with regards to the percentage of type I fibres containing high glycogen stores (*P* > 0.05; Fig. 3b*)*.

Rats in the CS group had a significantly lower percentage of type I fibres than both the DIT group (*P* < 0.005) and the DARE group (*P* < 0.05) (Fig. 3c*)*. The DCT animals also had fewer type I fibres than the DIT animals (*P* < 0.05; Fig. 3c).

### Type IIa fibres: IMCL stores, muscle glycogen stores, and total muscle percentage

There were no significant differences observed between all groups with regards to the percentage of type IIa fibres containing high IMCL stores (*P* > 0.05; Fig. 3d*)*.

Rats in the DIT group were found to have a higher percentage of type IIa fibres with high glycogen stores than rats in the CS group (*P* < 0.0001; Fig. 3e*).*

There were no significant differences observed with regards to the overall percentage of type IIa fibres analyzed between all groups (*P* > 0.5; Fig. 3f*)*.

### Type IIb fibres: IMCL stores, muscle glycogen stores, and total muscle percentage

There were no significant differences observed between all groups with regards to the percentage of type IIb fibres containing high IMCL stores (*P* > 0.05; Fig. 3g*)*, type IIb fibres containing high glycogen stores (*P* > 0.05; Fig. 3h*),* or the overall percentage of type IIb fibres analyzed (*P* > 0.05; Fig. 3i*).*

### Overall muscle fibre profile

**Fig. 4:**
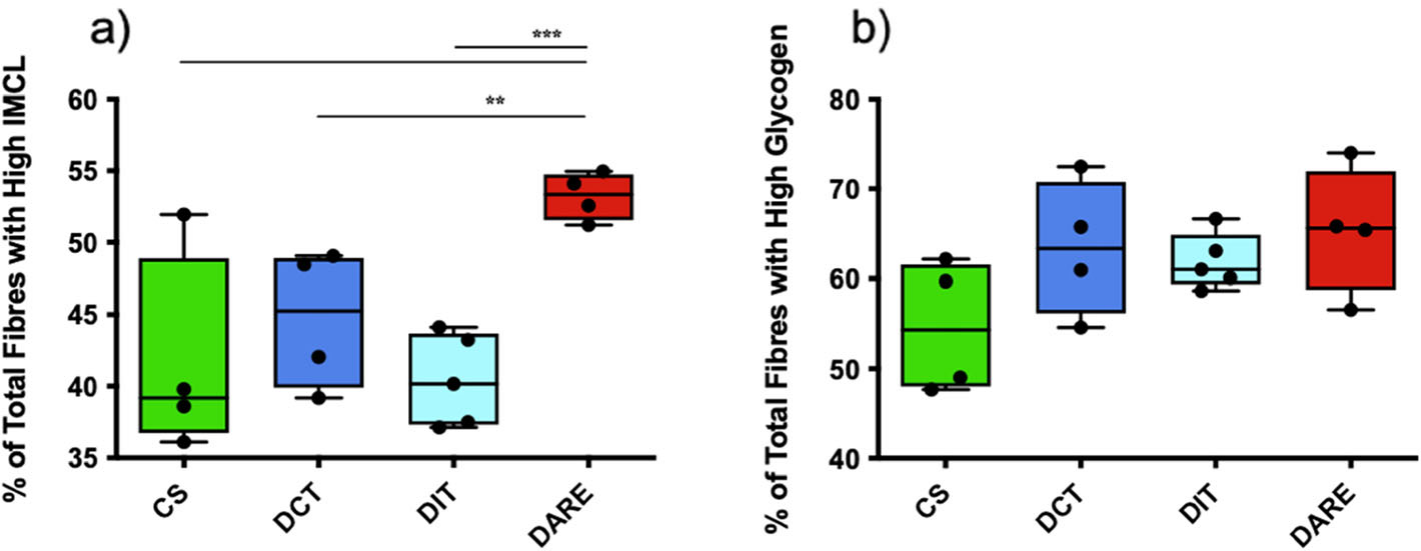
The whole-muscle metabolic profiles of 3630 muscle fibres from 17 male rats across four groups. The four groups are Control Sedentary (CS); diabetic with conventional insulin therapy (DCT); diabetic with intensive insulin therapy (DIT); and diabetic with combined exercise training and conventional insulin therapy (DARE). **a** The percentage of total fibres across all fibre types identified as containing high neutral IMCL stores via Oil Red O staining. **b** The percentage of total muscle fibres across all fibre types identified as containing high glycogen stores via glycogen periodic acid Schiff staining. All data are expressed as mean ± SEM. ** denotes *P* = 0.0094; *** denotes *P* = 0.0002. Note that the DARE group had significantly more high-IMCL containing fibres than each of the other groups, which is indicative of a well-documented result of exercise training known as the athlete’s paradox. Taken in conjunction with Fig. 3, it appears that this phenomenon occurs as a result of changing IMCL storage specifically within type I oxidative fibres

The rats in the DARE group had a greater percentage of high-IMCL containing muscle fibres than every other group (CS *P* < 0.0005; DCT *P* < 0.01; DIT *P* < 0.001) (Fig. 4a).

There were no significant differences observed between all groups regarding the percentages of total muscle fibres containing high glycogen content *(P* > 0.05; Fig. 4b*).*

## Discussion

It is well known that both T1DM and exercise place unique energetic demands upon skeletal muscle as a whole; however, the mechanisms by which individual muscle fibre types adapt to meet the combination of these demands remain to be understood. Previous research examining diabetic skeletal muscle health and function have examined whole muscle physiology, rather than investigating the differences between individual fibre types. Given that skeletal muscle fibres vary drastically in their metabolic profiles, their relative energy substrate usage, and their response to exercise, the purpose of this study was to determine how a combined endurance and resistance exercise training regimen would compare with intensive insulin therapy, the modern standard of care for patients with T1DM. Specifically, this study examined how T1DM impacted muscle fibre composition and energy substrate storage, and how these transitions adapted following a 12-week exercise intervention.

Results from the current investigation have demonstrated that only the type I muscle fibres showed any significant changes in IMCL storage. Moreover, the DIT group had a significantly lower percentage of type I high-fat fibres in comparison to CS and DCT (Fig. 3a*)*. While the DARE group had significantly fewer type I high-fat fibres than CS, there were significantly more type I fibres with high IMCL content than those in the DIT group and did not differ from DCT (Fig. 3a*)*. While this finding did not support the hypothesis that exercise-training would reduce the accumulation of fat content in diabetic skeletal muscle, it is plausible that the DARE animals benefited from a fibre-specific shift in metabolic fuel storage to accommodate the energetic demands of regular exercise. Indeed, it has been documented that exercise in non-diabetic patients leads to increases in IMCL storage [54]. Often referred to as the Athlete’s Paradox, this phenomenon in which exercise training leads to an increase in IMCL is often accompanied by improved metabolic indicators, such as improved insulin sensitivity. In further support of these findings, trained healthy participants have also been shown to increase their IMCL storage primarily in type I fibres, which are well equipped to handle this burden, thereby allowing the muscle to benefit from the increased IMCL stores [55].

Similar levels of IMCL content between DARE and DCT may be due to the chronically elevated levels of blood glucose (9–15 mM) between these two groups. It has been reported that increases in fat accumulation in skeletal muscle are proportional to increases in elevations in blood glucose levels [56]. Further, it has been shown that hyperglycemia-induced skeletal muscle fat levels are reversed following normoglycemia [56]. These findings would support a mechanism by which the DIT animals demonstrated a significant reduction in IMCL stores in comparison to DARE and DCT. Chronically hyperglycemic animals have been shown to demonstrate increased myocellular transmembrane lipid transporter cluster of differentiation 36 (CD36) [22, 36], accompanied by changes in serum non-esterified fatty acids levels [36] and decreased mitochondrial citrate synthase [22] and β-oxidation activity [36]. Indeed, it has been shown that increases in CD36 in hyperglycemic animals leads to elevations in fat transmembrane transport and accumulation in skeletal muscle [57]. In a previous report examining the same population of rodents, it was demonstrated that DARE led to a decrease in CD36 and an increase in citrate synthase activity in skeletal muscle to a greater extent than aerobic exercise training alone [22]. Despite these favourable metabolic alterations, elevated levels of lipid staining were evident in skeletal muscle of DARE animals in comparison to aerobic exercise alone [22]. It is not clear why DARE may lead to differential changes in fat content in skeletal content; however, importantly insulin sensitivity was elevated in comparison to aerobic exercise trained animals. This uncoupling of fat content and insulin sensitivity would suggest that exercising patients with T1DM exhibit the “athlete’s paradox,” whereby increases in lipid-oxidation capacity occur concomitantly with greater lipid storage. Moreover, it has been reported that insulin sensitivity is negatively correlated with IMCL content in untrained subjects, while the elevated IMCL content of exercised-trained subjects actually predicted high insulin sensitivity [58].

The negative impact of increased fat accumulation in skeletal muscle may be related to the density and/or size of the lipid droplet and its proximity to the mitochondria, as well as its subcellular pool (subsarcolemmal versus intramyofibrillar), though lipid droplet location was outside the scope of this study, and future research efforts should use techniques more suited for accurate determination of IMCL subcellular location. It has been reported that endurance-trained diabetic individuals exhibited elevated levels of skeletal muscle IMCL levels in comparison to untrained diabetic individuals due to increases in lipid droplet density (not lipid droplet size) [59]. Further, consistent with the current findings these increases in lipid density were specific to type I fibres only and unchanged in type II fibre types [59]. He et al. [60] reported that IMCL does not decrease in response to exercise in obese individuals; however, lipid within muscle is dispersed into smaller droplets which in turn are correlated to improved insulin sensitivity. Consistent with the above reports it is well accepted that IMCLs are primarily contained and used within type I muscle fibres, a conclusion which is supported by the observation that IMCL content is three times higher in type I oxidative fibres than in type IIb glycolytic fibres [61]. Moreover, type I muscle fibers appear to be more insulin sensitive than type II fibers [62]. This suggests that lipid toxicity (and by extension lipid metabolism-induced insulin resistance) is more closely associated with type I muscle fibres than type II fibres [63]. Upon examining the overall muscle fibre profile of the four study groups, this study identified significant differences in the percent of total fibres that stained as type I fibres between the various groups (Fig. 3c), but no differences between the relative percentage of type IIa (Fig. 3f) or type IIb fibres (Fig. 3i). The data from the current study have shown that the only changes in IMCL storage in T1DM occur within the type I fibres, and therefore any change in lipid-based insulin resistance would likely stem from the differences found within these fibres.

The only significant differences observed in muscle glycogen storage occurred between CS and DIT type IIa fast-twitch oxidative fibres (Fig. 3e*)*. Previous studies have found that whole-muscle glycogen storage did not change significantly between intensive insulin-treated T1DM patients and healthy controls [64, 65], although these studies did not distinguish between fibre types. The current study also examined muscle glycogen content without mapping either energy substrate to specific fibre types, and found no differences observed regarding whole muscle glycogen stores between groups (Fig. 4b). In support of the latter findings, it has been reported that glycolytic and oxidative enzyme activity have been shown to increase following exercise training; however, whole muscle glycogen storage remained unchanged, pre-training and post-training [64]. The reasons why DIT led to increases in glycogen content in a fibre-specific manner are unclear, but it is likely to do with the elevated levels of intensive insulin treatment in the animals. Type II fibres have higher stores of glycogen than type I fibers [66], which in the presence of hyperinsulinemia may lead to heightened glucose uptake in these fibres. It has been reported that hyperinsulinemia in the absence of hyperglycemia lead to greater increases in the uptake of glucose in gastrocnemius muscle (IIa > IIb > I fibers) versus soleus (I > IIa > IIb fibers) [67]. More work is needed to understand the mechanisms leading to elevated glycogen content in T1DM and its functional impact on skeletal muscle health, particularly as it pertains to intensive insulin therapy (IIT).

### Study strengths and limitations

The present study has several important strengths. Firstly, homogenous groups of T1DM rats performed a combination of standardized aerobic and resistance exercise sessions that is reflective of the recommended exercise training modality prescribed to patients with T1DM [68–71]. Further, in this study T1DM rats underwent two separate insulin treatment regimens that resulted in blood glucose levels reflective of the T1DM patient population with and without prescribed exercise. Lastly, the study design afforded the deeper investigation into the T1DM-related alterations in skeletal muscle fibre type and metabolic content and how regular exercise may mitigate the negative changes in muscle health associated with this disease.

The main limitation of the current study was the relatively small sample size, which may have affected the ability to detect statistically significant changes in fibre type and metabolic content. Specifically, the low sample size may have limited the ability to determine changes in glycogen content in type I and type II fibres between groups. Additionally, a non-T1DM exercise-trained group was not included in the study design, which did not allow the comparison of the muscle specific benefits associated with exercise in T1DM animals versus otherwise healthy non-diabetic animals. Though the current study utilises an established rat model of both well-controlled and poorly-controlled T1DM, it is a pre-clinical animal trial and therefore these results provide insight only into the role of exercise in skeletal muscle health in clinically-treated patients with T1DM. Finally, the animals were not monitored for food intake, and changes in feeding patterns could have metabolic repercussions that may be reflected in the skeletal muscle examined in the current study.

## Conclusions

The current results demonstrate that changes in IMCL and skeletal muscle glycogen content in response to T1DM and combined exercise are fibre-type specific. The percentage of type I skeletal muscle fibres was increased in exercise-treated and IIT-treated animals. While IIT led to a reduction in IMCL stores in type I fibres, this reduction in IMCL was not evident in exercise-treated animals. Type IIa and IIb fibres showed no changes in IMCL, while type IIa demonstrated an increase in glycogen content following IIT. Taken together, these findings would indicate that alterations in skeletal type I muscle fibre proportion and fuel content are evident following IIT and combined exercise training. While IIT reduces IMCL stores, combined exercise training leads to an increase in preferential fat storage to oxidative type I fibres without significant glycogen storage changes. Significant alterations in IMCL content primarily in type I fibres in response to varied insulin regimens and exercise stimulus suggest that lipid-related myopathies in T1DM, such as lipotoxicity-related insulin resistance, may occur in a muscle-fibre-dependent manner.

Insulin resistance is a major risk factor for long-term morbidity and mortality in patients with T1DM, and it has been linked to the improper storage and lipotoxic accumulation of certain IMCL species (primarily diacylglycerol; DAG) within the skeletal muscle. The current study demonstrates that this accumulation of IMCLs, and their subsequent reduction with exercise, does not occur uniformly across all three muscle fibre types. Rather, significant alterations in IMCL content occurred in the type I slow-twitch oxidative fibres only. From a clinical perspective, this finding suggests that future treatment strategies to mitigate insulin resistance development in patients with T1DM should include exercise programs specifically tailored to stimulate metabolic improvements in lipid flux and storage within the type I oxidative fibre. With the ability to strategically implement exercise therapy in a way that both complements a patient’s insulin treatment and improves type I myofiber metabolism, clinicians will be able to manage T1DM progression in a way that reduces the impact of insulin resistance through the prescription of regular exercise.

## Supplementary Information

**Additional file 1.** Quantification Example.

## Data Availability

The datasets during and/or analysed during the current study available from the corresponding author on reasonable request.

## Abbreviations

A: Aerobic
ANOVA: Analysis of variance
ATP: Adenine triphosphate
BG: Blood glucose
CD36: Transmembrane glycoprotein cluster of differentiation 36
CIT: Conventional insulin treatment
CS: Control sedentary
CVD: Cardiovascular disease
DAG: Diacylglycerol
DARE: Diabetic combined (aerobic/resistance) exercise training treated with conventional insulin
DCT: Diabetic sedentary treated with conventional insulin
DIT: Diabetic sedentary treated with intensive insulin
H/E: Hematotoxylin/eosin
IIT: Intensive-insulin treatment
IMCL: Intramyocellular lipid
IR: Insulin resistance
NDPL: Neutral density polarized lens
R: Resistance
STZ: Streptozotcin
T1DM: Type 1 diabetes mellitus
T2DM: Type 2 diabetes mellitus
VO_2_max: Maximal oxygen consumption
