# Supplementary material for: The effects of exercise training versus intensive insulin treatment on skeletal muscle fibre content in type 1 diabetes mellitus rodents
Source: Lipids Health Dis. 2021 Jul 6;20:64. doi: 10.1186/s12944-021-01494-w (PMC8262066; doi:10.1186/s12944-021-01494-w)
Supplement: Supplementary file 1 — Additional file 1. Quantification Example. [file 12944_2021_1494_MOESM1_ESM.docx]

**Additional file 1: Quantification Example**


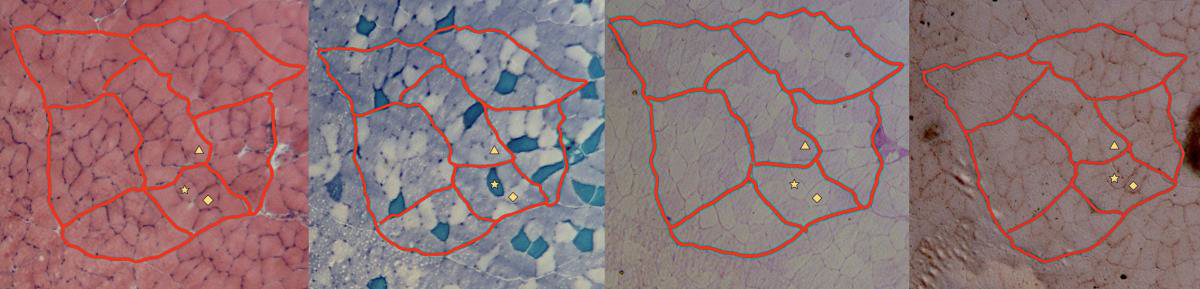


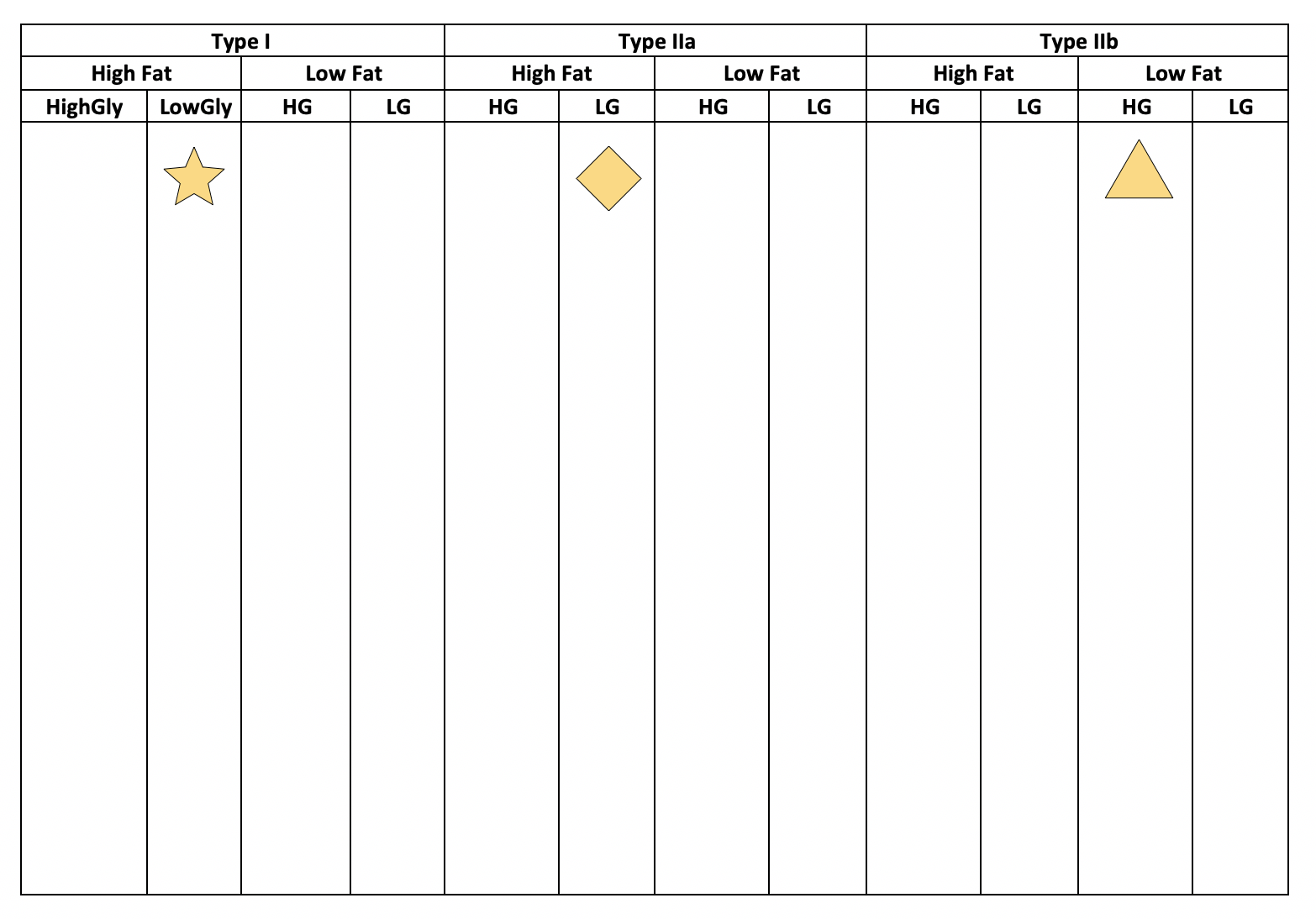


Quantification consisted of identifying the same fibre across four stains (hematoxylin & eosin, metachromatic ATPase, glycogen periodic acid Schiff, oil red O) ensuring there was a clear distinction between high/low and fibre type, and then using a tally sheet to collect data.
